# A thank you note to our peer reviewers (2024)

**DOI:** 10.1002/jia2.70017

**Published:** 2025-07-15

**Authors:** Kenneth H. Mayer, Annette H. Sohn, Marlène Bras

**Affiliations:** ^1^ The Fenway Institute and Harvard Medical School Boston Massachusetts USA; ^2^ TREAT Asia, amfAR ‐ Foundation for AIDS Research Bangkok Thailand; ^3^ IAS – the International AIDS Society Geneva Switzerland

1

The *Journal of the International AIDS Society* (JIAS) would like to express our gratitude to the peer reviewers who contributed to reviewing articles for the journal in 2024, and to those senior scientists who have mentored early career researchers in reviewing.

Your time and expertise are crucial to upholding the quality of this publication, and we are thankful for your engagement.

We also wish to extend our appreciation to the JIAS Editorial Board members, Deputy Editors, Statistical Committee and Ethical Committee members for their valuable contributions in assessing and reviewing submitted articles.

Kenneth Mayer, co‐Editor‐in‐Chief

Annette Sohn, co‐Editor‐in‐Chief

Marlène Bras, Executive Editor

Aaloke Mody

Abhishek Reddy Mogili

Abiye Kalaiwo

Adiatma Yudistira

Aditya Singh

Akarin Hiransuthikul

Akshay Sharma

Alana Sharp

Alana T Brennan

Alex de voux

Alexandra Calmy

Alison Castle

Alison Kutywayo

Alison Roxby

Alissa Davis

Aliza Monroe‐Wise

Allanise Cloete

Amit Achhra

Amy Medley

Amy Zheng

Andrew David Forsyth

Andrew Mujugira

Andrew Wiznia

Angela Bengtson

Angela Colbers

Angela Hutchinson

Ann Gottert

Anna Bershteyn

Anna Bowring

Anna Downie

Anna Klicpera

Anna Yeung

Annaliese M. Limb

Anne Neilan

Antons Mozalevskis

Apostolos Beloukas

Ariane van der Straten

Arman Oganisian

Arturo M. Ongkeko

Assel Terlikbayeva

Audrey Pettifor

Ava Avalos

Ayako Fujita

Barbara A. Friedland

Beatrice Wamuti

Benjamin Brown

Benjamin Ryan Phelps

Bindiya Meggi

Bluma Brenner

Brendan Harney

Brian Honermann

Brian T Foley

Bridget Haire

Bright Ofori

Brittany Moore

Brooke E Nichols

Bruno Spire

Caitlin E. Kennedy

Carlos Cáceres

Carlos E. Rodriguez‐Diaz

Carol Strong

Caroline A. Bulstra

Caroline DeSchacht

Carolyn Chu

Caryl Feldacker

Catherine Godfrey

Catherine Koss

Catherine Orrell

Catherine Verde Hashim

Cedric Bien‐Gund

Chanda Mwamba

Charles Ssonko

Chen Seong Wong

Chenai Mlandu

Chenglin Hong

Cheryl Case Johnson

Chloe A. Teasdale

Chris Collins

Christian Grov

Christian Stillson

Christiana Noestlinger

Christopher James Hoffmann

Connie Celum

Craig J Heck

Cristina Pimenta

Cuc Tran

Curtis Chan

Dadong Wu

Darrell Tan

Dawn Goddard‐Eckrich

Deanna Kerrigan

Debbie Humphries

Debrah Boeras

Denton Callander

Diego Cecchini

Dion Carleen Allen

Divya Ramani Bhamidipati

Dominic Reeds

Dominique Medaglio

Donn Colby

Dumbani Kayira

Dunstan Achwoka

Dusita Phuengsamran

Dvora Joseph Davey

Ebiere Clara Herbertson

Edmon Obat

Edsel Salvana

Edy Nacarapa

Eiichi N Kodama

Elijah Kakande

Elise Lankiewicz

Elizabeth Ellen Tolley

Elliot Raizes

Elwin Wu

Elzette Rousseau

Emilia Jalil

Emily Hyle

Emma Kalk

Emmanuel S Baja

Enrique Saldarriaga

Erin Ferenchick

Eugenia L. Siegler

Fekadu Adugna

Felipe Cazeiro

Felix Abuna

Frances Mary Cowan

Francois Cholette

Francoise Renaud

Fumiyo Nakagawa

Gabriel Chamie

Gabriela Patten

Gemma Oberth

Geoff Garnett

Gerhard Theron

Godwin Anguzu

Goodluck Willey Lyatuu

Gregory F. Guzauskas

Gregory M Lucas

Habib Ramadhani

Hannah Yellin

Hanne Zimmermann

Hannock Tweya

Haroon Moolla

Hayley Cunningham

Helen Bygrave

Heli Harvala

Hellen Moraa

Homaira Hanif

Homero E del Pino

Hyman Scott

Ian Armstrong

Isaac I. Bogoch

Isaac Kimera

Ismail Lawal

Iyeseun Olusola Asieba

J Peter Figueroa

Jacob Bleasdale

Jacqueline Pienaar

Jake Michael Pry

James Ayieko

James Carlucci

James Moody

Jane Kabami

Janet Seeley

Jason J Ong

Jason Michael Bacha

Jasper S. Lee

Jean‐Pierre Routy

Jeb Jones

Jennifer Fusco

Jennifer J Carroll

Jennifer M Belus

Jennifer Manne‐Goehler

Jennifer Sherwood

Jeremy Ross

Jessica E Haberer

Jiawei He

Jienchi Dorward

Jill T Owczarzak

Jillian Pintye

Jirair Ratevosian

Jiun‐Hau Huang

Joan Tallada

Joanna Busza

Joanne M Carson

Joep J. Van Oosterhout

John Joska

John M Humphrey

Jonathan M. Grund

Jonathan Murray King

Jonathan Odumegwu

Joseph Larmarange

Joseph Matovu

Jose‐Ramon Blanco

Joy Nakato

Julia Clare Dettinger

Julia Marcus

Julia Rohr

Julian Adong

Julie Dumond

Julie Pulerwitz

Julie V. Sudovec‐Somogyi

Julien Brisson

K Rivet Amico

Kadija Muse Tahlil

Karin Hatzold

Karine Dube

Karl Technau

Kassem Bourgi

Kate Barnighausen

Kate Wilson

Katharine Kripke

Katie Williams

Katrina Frances Ortblad

Kelika Konda

Kellie Freeborn

Kenneth Mugwanya

Keosha Bond

Kevin O'Callaghan

Khadijat Adeleye

Kim AGJ Romijnders

Kimberly Hook

Kimberly Powers

Kiran Paudel

Koharu Loulou Chayama

Kombatende Sikombe

Kristefer Stojanovski

Kristin Baltrusaitis

Kudzai Kumbasha‐Hlahla

Lai Peng Ho

Lao‐Tzu Allan‐Blitz

Lara Lorenzetti

Larry W. Chang

Latasha Elopre

Laura Packel

Lauren Cirrincione

Lauren M. Hill

Leigh M. McClarty

Lena Andersen

Leonardo Rabena Estacio

Leslie A Enane

Lila A Sheira

Lindsey de Vos

Lindsey Marie Filiatreau

Linxuan Wu

Lisa Frigati

Lisa Lazarus

Lise Jamieson

Liudmyla Legkostup

Lora Sabin

Louis Masankha Banda

Lucia Taramasso

Lucy Andere Chimoyi

Luh Putu Lila Wulandari

Lynda Stranix‐Chibanda

Lynn Matthews

Lynne Wilkinson

Magdalene Walters

Mahoudo Bonou

Maitreyi Sahu

Makella Coudray

Makobu Kimani

Manogar Siregar

Manya Magnus

Maria Grazia Lain

Maria Mazzitelli

Marie Ballif

Marie‐Claude Lavoie

Mariet Benade

Marija Pantelic

Marina Klein

Mark Marzinke

Mark van den Elshout

Martin Brizuela

Martin Muddu

Mateo Prochazka Núñez

Mathieu Maheu‐Giroux

Matinatsa Mugore

Matthew A. Spinelli

Matthew D Hickey

Matthew L Romo

Matthew Pitman

Maxime Charest

Maxime Inghels

Megan Hansen

Melissa Turner

Meng Li Chong

Mercelline Ogolla‐Onyando

Meredith Clement

Michael Evangeli

Michael Herce

Michael Martin

Michael Thomson

Michelle Choy

Ming Jian Ni

Minh X. Nguyen

Miran Šolinc

Mitchell Warren

Molly Franke

Monisha Sharma

Moussa Sarr

Musonda Simwinga

Mwelwa Muleba Phiri

Myron Cohen

Nanina Anderegg

Nao Yamamoto

Nasheed Moqueet

Natella Rakhmanina

Nathan P Ford

Natsayi Chimbindi

Neetha Morar

Ngai‐Sze Wong

Nicole Bellows

Nicole Perreras

Nimasha B Fernando

Nixon Amuomo

Nkgomeleng Lekodeba

Noah Mancuso

Nobubelo Ngandu

Nok Chhun

Nomcebo Oratile Mokgethi

Olivia van Gerwen

Oluwaseun Abdulganiyu Badru

Oluwaseun Ayoola Ojomo

Pablo Rojo Conejo

Pamela Bachanas

Pamela Payne‐Foster

Pat Flynn

Patricia Atieno Ong'wen

Patrick Castillo Eustaquio

Patrick Ryscavage

Paula Vaz

Pearl Anne Ante‐Testard

Pedro Botti Carneiro

Pengfei Wang

Peter A Newman

Peter Anderson

Peter F. Rebeiro

Peter Godfrey‐Faussett

Peter Saxton

Philip John Smith

Pich Seekaew

Prakash Ganesh Ganesh

Primrose Matambanadzo

Prince Agyeman

Qiang Xia

Quarraisha Abdool Karim

Rami Kantor

Ramnath Subbaraman

Rayner K.J. Tan

Rebecca K Fielding‐Miller

Rebecca Martinez

Reena Rajasuriar

Rena Patel

Renata Arrington Sanders

Renee Heffron

Rhonda White

Ricardo Macedo Abrantes

Richard Gray

Richard J. Jefferys

Risa Hoffman

Robert Remien

Roland Merchant

Ronnie M Gravett

Rosa Bologna

Rose Pollard Kaptchuk

Roseline Iberi Aderemi‐Williams

Roxanne Kerani

Rudy Patrick

S Wilson Cole

Samir Gupta

Samuel Kalibala

Santhi Hariprasad

Sara Landers

Sarah E. Rutstein

Scott Dryden‐Peterson

Scott McClelland

Sharmistha Mishra

Shawn Malone

Sheela Shenoi

Simona Sacuiu

Siobhan Malone

Siphamandla Bonga Gumede

Sita Lujintanon

Solange Baptiste

Sophia Rein

Sophie J S Pascoe

Stacey Hannah

Sten H Vermund

Stephane Wen‐Wei Ku

Stephanie Marie Davis

Stephen Okoboi

Stephen Ramos

Steve Shoptaw

Steven Deeks

Steven Safren

Sue M Napierala

Susan Marie Graham

Susanne Doblecki

Swarnali Goswami

Sydney Rosen

Sylivia Nalubega

Taurayi A. Tafuma

Tamsin Kate Phillips

Tara McCrimmon

Tasnim Azim

Tatiana Tarasova

Tetiana Kiriazova

Thanyawee Puthanakit

Thibaut Davy‐Méndez

Tim Brown

Tinashe Grateful Rufurwadzo

Tinne Gils

Todd M Pollack

Tonia Poteat

Trudy Leong

Tyler Wray

Unmesha Roy Paladhi

Valerie Yelverton

Vamsi Vasireddy

Vanda Cota

Varsha Srivatsan

Venkatraman Chandra‐Mouli

Victoria Simms

Virginia A. Fonner

Virginia Macdonald

Vita W Jongen

Webster Mavhu

Willem DF Venter

William Reidy

Win Han

Xin Niu

Yael Hirsch‐Moverman

Yagai Bouba

Yan Nee Gan

Yana Kirey‐Sitnikova

Ye Kyaw Aung

Yunia Mayanja

Zaynab Essack

